# Sustained Selective Attention to Competing Amplitude-Modulations in Human Auditory Cortex

**DOI:** 10.1371/journal.pone.0108045

**Published:** 2014-09-26

**Authors:** Lars Riecke, Wolfgang Scharke, Giancarlo Valente, Alexander Gutschalk

**Affiliations:** 1 Department of Cognitive Neuroscience, Faculty of Psychology and Neuroscience, Maastricht University, Maastricht, The Netherlands; 2 Department of Child and Adolescent Psychiatry, Psychotherapy and Psychosomatics, University Hospital, RWTH Aachen University, Aachen, Germany; 3 Department of Neurology, Ruprecht-Karls-Universität Heidelberg, Heidelberg, Germany; Harvard Medical School/Massachusetts General Hospital, United States of America

## Abstract

Auditory selective attention plays an essential role for identifying sounds of interest in a scene, but the neural underpinnings are still incompletely understood. Recent findings demonstrate that neural activity that is time-locked to a particular amplitude-modulation (AM) is enhanced in the auditory cortex when the modulated stream of sounds is selectively attended to under sensory competition with other streams. However, the target sounds used in the previous studies differed not only in their AM, but also in other sound features, such as carrier frequency or location. Thus, it remains uncertain whether the observed enhancements reflect AM-selective attention. The present study aims at dissociating the effect of AM frequency on response enhancement in auditory cortex by using an ongoing auditory stimulus that contains two competing targets differing exclusively in their AM frequency. Electroencephalography results showed a sustained response enhancement for auditory attention compared to visual attention, but not for AM-selective attention (attended AM frequency vs. ignored AM frequency). In contrast, the response to the ignored AM frequency was enhanced, although a brief trend toward response enhancement occurred during the initial 15 s. Together with the previous findings, these observations indicate that selective enhancement of attended AMs in auditory cortex is adaptive under sustained AM-selective attention. This finding has implications for our understanding of cortical mechanisms for feature-based attentional gain control.

## Introduction

How can we hear out a sound in an auditory scene? According to contemporary views [1], [2], the extraction of a sound of interest from a mixture is facilitated by directing one's attention toward a distinctive feature of that sound, as this leads to selective enhancement of that feature and temporally coherent features in the cortex relative to unattended features. Evidence for such a top-down, feature-based gain control mechanism comes from several human brain studies showing that selective attention to a specific tone frequency or a specific location enhances neural responses to sounds that comprise the attended frequency or originate from the attended location, respectively [3], [4], [5], [6], [7], [8], [9]. Thus, attentional gain control seems to operate on various sound features in the cortex, including tone frequency and sound location.

Recently, this idea has been extended to amplitude modulation (AM), i.e., the temporal envelope of the sound waveform. It has been shown that selective attention to an AM sound may enhance cortical responses synchronized with the AM (the auditory steady-state response, SSR), compared with selective attention to a differently modulated, competing sound [10], [11], [12], [13] or to visual input [14], [15], [16], [17], [18], [19], [20]. Considering that AM has been suggested to be encoded in AM-frequency specific channels [21], [22] in the auditory cortex (AC) [23], [24] and earlier processing stages [25], [26], [27], [28], these findings may suggest that attentional gain control operates on temporal AM representations in AC.

A limitation of these studies is that the attended sound could be distinguished from the unattended sound based on not only AM, but also other sound features, such as carrier frequency or location. Thus, it remains unclear whether the observed response enhancements reflect selective attention to AM or to other sound features that may have been enhanced, e.g., through tonotopic or location-specific representations that were captured by the SSR due to their temporal coherence with the AMs [2]. Moreover, most studies used relatively short sounds in the range of a few seconds or less and did not investigate changes in response enhancement over time. Thus, it remains unclear whether AM-specific attentional gain control operates stably over intervals of several tens of seconds [18]. Finally, most studies focused on rapid AMs (AM frequencies of 20 Hz or higher), while comparatively little is known about gain control for slower AMs in the range of a few Hz [12], [13] although the latter are crucial for speech comprehension [29]. The few studies that used relatively long speech sounds (spoken sentences) [30], [31], [32] found sustained speaker-selective attentional enhancement in AC, even when the competing sounds originated from the same location, had similar frequencies, or produced similar peripheral excitation patterns. As for the other studies, it is uncertain whether listeners in these studies attended exclusively to the slow AMs in the speech signals or to other distinctive sound features, such as the timbre of the voice or the size of the resonance body.

The goal of the present study was to address the previous limitations and to investigate gain control in human cortex based on attention to slow AM frequencies alone over a long interval. We tested whether selective listening to one of two competing periodic AM frequencies is accompanied by selective enhancement of the temporal representation of that AM frequency in AC (compared with the representation of the competing AM frequency, which was applied to the same tone carrier at the same location). To that end, we first identified the time-locked auditory cortical representation of the individual AM frequencies using the SSR measured with scalp electroencephalography (EEG). We then characterized this temporal AM-frequency representation together with participants' perception under different attentional (but otherwise similar) conditions induced by behavioral tasks that required either sustained selective listening to one or the other AM frequency, or visual attention.

Our principal finding is that sustained AM-selective attention does not induce sustained response enhancement for the attended AM compared with the ignored AM. Although we observed an initial trend toward AM-selective attentional response enhancement, overall the response to the ignored AM frequency was enhanced.

## Materials and Methods

### Participants

Fourteen paid volunteers (eight females, ages 18–39 years) with no reported hearing, vision, or motor problems participated in the study after providing written informed consent. Ethical approval was obtained from the local ethics committee (*Ethische Commissie Psychologie*) of the Faculty of Psychology and Neuroscience of Maastricht University.

### Stimuli

**Figure 1:**
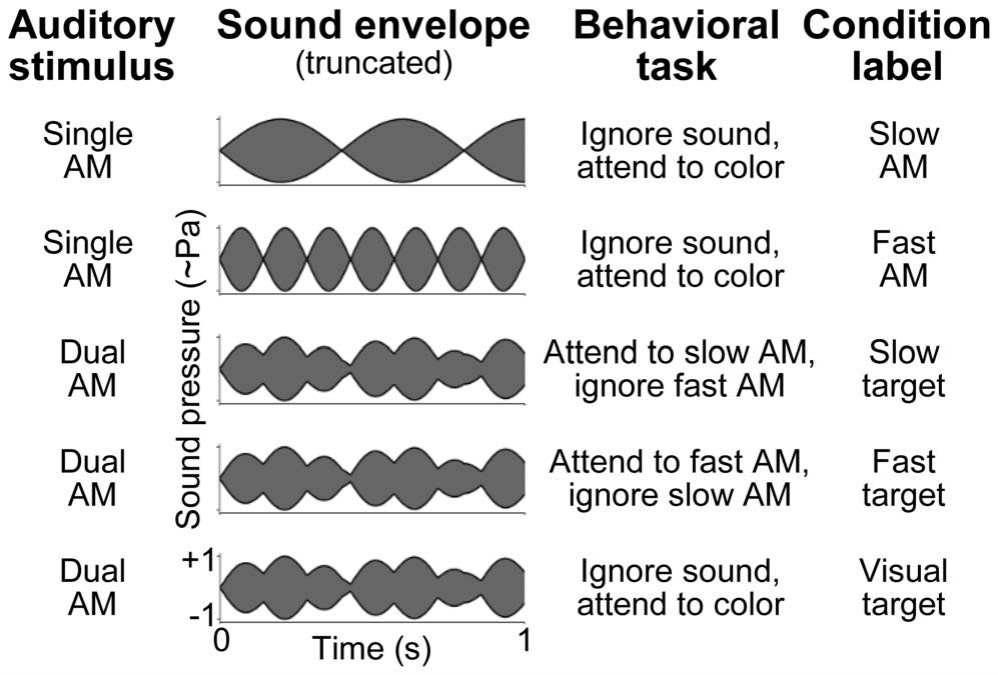
Auditory stimuli, behavioral tasks, and experimental design. Simple auditory rhythms were generated by applying either a slow or fast AM to a fixed pure tone carrier (rows 1, 2). A polyrhythm was generated by mixing these single rhythms, i.e., both AMs were applied to the same carrier and then added (rows 3–5). The stimuli had a duration of 55 s and were presented during selective auditory and visual attention tasks (third column), which served to draw participants' attention to the slow or fast rhythm (rows 3, 4) or away from auditory input (rows 1, 2, 5). The single-AM conditions were used to identify the time-locked neural representation of the individual AMs. The dual-AM conditions were used to test whether this temporal AM representation was selectively enhanced during sustained selective attention to/away from a specific AM (in the absence of acoustic stimulus differences and location or pitch cues for streaming).

Figure 1 illustrates the waveforms of the auditory stimuli. Stimulus duration was set to 55 s to allow studying changes of both perception and AM representation over a long interval. The stimuli contained either a single AM tone (single-AM stimuli; see Figure 1, upper two rows) or two AM tones of the same carrier frequency (dual-AM stimulus; see Figure 1, lower three rows) [33], [34], [35]. The single-AM stimuli were generated by multiplying a 930-Hz sinusoidal carrier with a full-wave rectified full-amplitude sinusoidal modulator (modulation depth: 100%). The frequency of the rectified modulator was set to 2.5 Hz (*f_1_*) or 7 Hz (*f_2_*) to create what we will refer to as “slow AM” or “fast AM”, respectively. The dual-AM stimulus was generated by adding the slow AM and fast AM after setting amplitudes so that both AMs would appear equally salient (see below, section Procedure).

The specific modulation rates chosen had several advantages. Firstly, they allow studying neural correlates of syllable analysis [36] and speech comprehension [29]. Secondly, they evoke robust responses in the EEG power spectrum for pure tone carriers, i.e., strong and well-separated peaks at low harmonics [33], [37], [38], [39], [40], [41]. Thirdly, they are sufficiently different from each other to provide robust temporal cues for auditory stream segregation (referred to as “streaming” in the following) [42]. Finally, they are sufficiently similar and sufficiently slow to reduce spectral cues and pitch cues for streaming, because the side-bands that these modulations induce in the stimulus spectrum are not resolved in the excitation pattern of the auditory nerve [43].

The auditory stimuli were matched for peak amplitude. Starting phases of carrier and modulators were held constant throughout. Stimuli were sampled at a rate of 44.1 kHz with 16-bit resolution and presented binaurally at maximal 65 dB SPL using Presentation software (Neurobehavioral Systems), a Sound Blaster Live sound card (Creative Technology), a Soundcraft EXF8 mixer (Harman), a P4050 amplifier (Yamaha), and two JBL Control 25 loudspeakers (Harman). The speakers were located in front of the participant in the left and right upper corner of the EEG recording chamber. The speaker-ear distance was approximately 1.5 m.

### Task and design

The experimental design involved the five conditions illustrated in Figure 1 (see labels in last column). They were defined by combining the three auditory stimuli (slow, fast, or dual AM) with selective auditory and visual attention tasks that served to draw the participants' attention either toward a specific AM or away from the auditory stimuli and keep participants in a stable alert state.

For the selective listening task, the dual AM was used as the auditory stimulus. Participants were instructed to focus their attention on either the slow or fast rhythm in the mixture, depending on the experimental condition (“slow target” condition or “fast target” condition, respectively), and ignore the concurrent “non-target” rhythm. They were further instructed to report their current percept whenever a rhythm became perceptually dominant over the other (when the initial percept had finished building up and, thereafter, in case a perceptual reversal occurred) by pressing a corresponding button. For the visual attention task, either one of the single AMs (“slow AM” condition or “fast AM” condition) or the dual AM (“visual target” condition) was used as the auditory stimulus, depending on the experimental condition. Participants were instructed to ignore this stimulus and focus their attention on the color of a fixation cross. The cross was presented in white on a black screen throughout all auditory and visual tasks, and in the visual task, it further changed its color to green, red, or blue for 200 ms at irregular times. Participants were instructed to report whenever the cross turned green or red in the visual task by pressing a corresponding button. Participants performed the tasks using their two index fingers on two buttons of a button box. The labels of the two buttons were “*slow*” and “fast” (for the *slow* target condition), “slow” and “*fast*” (for the *fast* target condition), or “green” and “red” (for the visual target condition), and they were shown above the cross in the center of the screen.

Individual trials lasted 80 s and contained three consecutive intervals. The first 5 s comprised a preparation interval, during which the cross and the button labels indicating the upcoming task lighted up. The subsequent 55 s comprised the task interval, during which an auditory stimulus was presented and the participant performed the task as indicated, while the cross and button labels remained visible. The final 20 s comprised a rest interval, during which only the cross remained visible for another 10 s followed by a blank screen. The order of trials was pseudorandomized so that each combination of two successive conditions appeared equally often in order to counterbalance possible long-term perceptual aftereffects of preceding modulations [44], [45], [46].

### Procedure

#### AM-saliency matching

Participants were seated in a comfortable chair in a sound-attenuating, electrically shielded booth. Saliency matches for the slow and fast AM were obtained using a sound level adjustment task as follows. Participants were presented with the dual-AM stimulus and asked to adjust the amplitude ratio of the slow and fast rhythm so that these rhythms would appear equally salient. The AM-saliency matches, defined as the average of ten measurements, revealed that participants scaled the amplitudes of the slow AM and fast AM on average to a ratio of 1∶1.3, indicating that they perceived the slow AM in the original dual-AM stimulus as more salient, in line with results on modulation detection [47], [48]. For the subsequent EEG experiments, the relative amplitudes in the dual-AM stimulus were set individually according to the obtained matches so that the two AMs would appear equally salient.

#### EEG experiments

Following AM-saliency matching, participants practiced the different tasks until they felt confident that they could perform them well. For the EEG measurements, they received further instructions to keep their gaze at the fixation cross, to relax, and to avoid motor activity other than button presses. They then underwent five blocks of EEG measurements, each comprising nine experimental trials and simultaneous EEG recordings, with self-terminated breaks in between blocks. In total, nine trials of each condition were presented and one hour of experimental EEG data was recorded. During debriefing, participants were asked to provide written report of their strategies for performing the tasks and rate their perception of a potential third “beating” rhythm in the dual AM conditions (*f_2_*-*f_1_*, the interaction of the individual AM rates in the peripheral auditory system) on a two-point scale.

#### EEG recording

EEG was recorded from 64 positions on the scalp in reference to the left mastoid, using Ag/AgCl electrodes (mounted in Easycaps, modified full 10%-system) and Neuroscan amplifiers that were decoupled from the audio system via optical fibers. Electrooculography was recorded below the left eye using an additional electrode. Interelectrode impedances were kept below 5 kΩ by abrading the skin. The EEG recordings were bandpass-filtered (cutoffs: 0.05 and 100 Hz, analog filter) and then digitized using a sampling rate of 250 Hz.

### Behavioral data analysis

Button responses in the auditory task were re-sampled at a rate of 1 Hz to create a time series of participants' reported dominant percept (which alternated between the target and the non-target) for each trial. Two measures were extracted from these on/off-series for each participant: First, after concatenating the trials, a perceptual dominance index was defined as the proportion of overall time that the participant reportedly perceived the target as dominant. Second, for each time point, the probability of perceiving the target was computed for each target condition (slow target, fast target) as the proportion of trials that the participant reported perceiving the target as dominant. The time series of this latter measure was used to identify the endpoint of the interval during which perceptual dominance initially built up [49] (by averaging across the two target conditions and across participants, fitting a sixth-order polynomial, and extracting the time point of the earliest curve slope reversal). Button responses in the visual task were considered as hits or false alarms, depending on whether the reported color did or did not match the actual color, respectively. Hit rates and false alarm rates were computed and transformed into *z*-scores that were then subtracted to obtain the sensitivity index *d*' [50]. On average, participants made 2.1±1.0, 2.4±1.3, and 4.0±0.02 button presses (mean ± s.d. across participants) per trial in the slow target, fast target, and visual target condition, respectively.

### Neural data analysis

**Figure 2:**
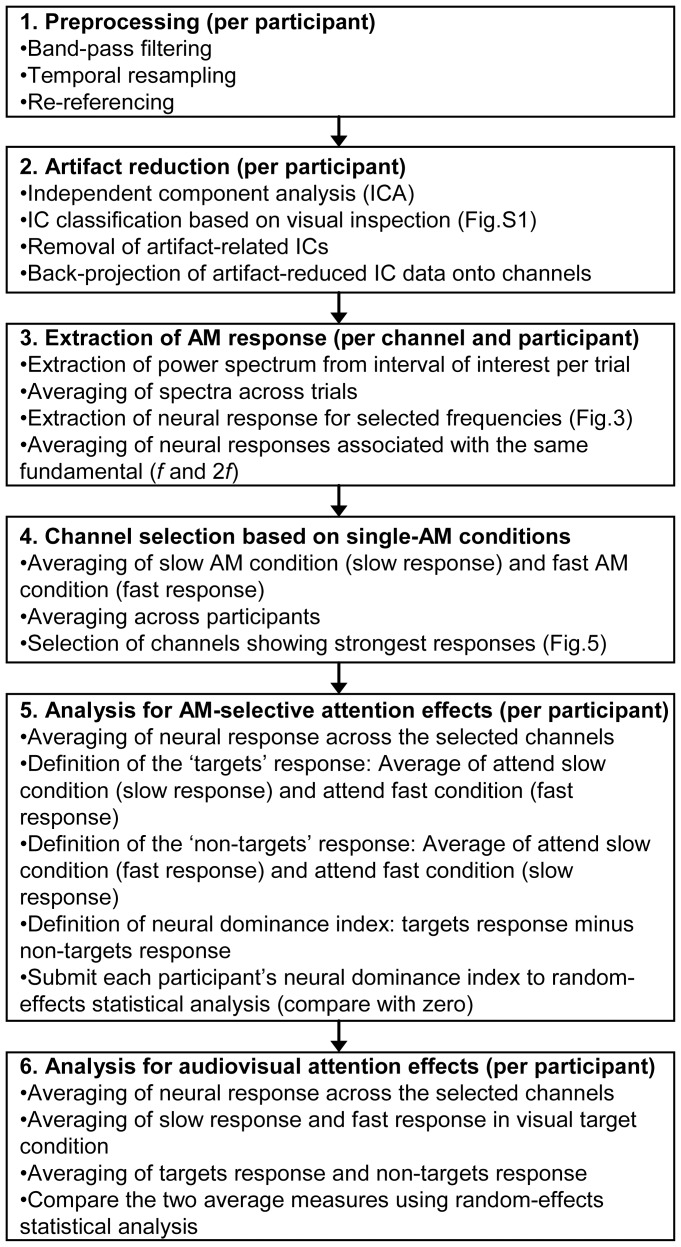
EEG data processing steps. The flowchart provides an overview of the main processing steps applied to the EEG data. Further details are provided in the main text (section Data analysis), Figure S1 (step 2), Figure 3 (step 3), and Figure 5 (step 4). Additional analyses are explained in the main text.

An overview of our EEG data analysis steps is provided in Figure 2.

#### EEG data preprocessing

EEG data were analyzed using the EEGLAB toolbox [51] and custom Matlab scripts. Data preprocessing involved band-pass filtering (cutoffs: 0.5 and 50 Hz, FIR filter), temporal resampling (sampling rate: 125 Hz), and re-referencing to an average reference (based on the mean activity of all channels). To reduce artifacts, the channel waveforms from each participant were first decomposed into a linear sum of 65 spatially fixed and maximally temporally independent components (ICs) using the extended Infomax ICA algorithm [52], [53]; for details see Figure S1. The main advantage of the ICA-based artifact reduction is that it allows the removal of repetitive artifacts without the need to reject entire data epochs [54], [55], [56]. Next, ICs resembling brain activity were separated from ICs resembling artifacts using visual inspection and standard criteria: ICs primarily accounting for eye movements or blinks were identified based on their far-frontal scalp distributions and irregular occurrence/timing across trials. Other artifact-related ICs, including those accounting for motor activity, were identified based on their non-dipolar scalp maps, flat activity spectra, and irregular occurrence/timing across trials [54], [56]. Finally, ICs deemed to resemble brain activity (on average 26±4 ICs, mean ± s.d. across participants) were recomposed and back-projected to yield artifact-reduced EEG channel waveforms.

#### Extraction of normalized neural response

Neural responses to AM were assessed using the SSR, which captures the magnitude of neural activity fluctuating at the AM frequency. Our frequencies of interest included the first two harmonics of the slow AM (*f_1_*: 2.5 Hz, 2*f_1_*: 5 Hz) and the fast AM (*f_2_*: 7 Hz, 2*f_2_*: 14 Hz), and the beat frequency (*f_2_*-*f_1_*: 4.5 Hz). Two arbitrary, stimulus-unrelated frequencies (the first two harmonics of both 2.2 Hz and 6.7 Hz) were further chosen to serve as control frequencies.

**Figure 3:**
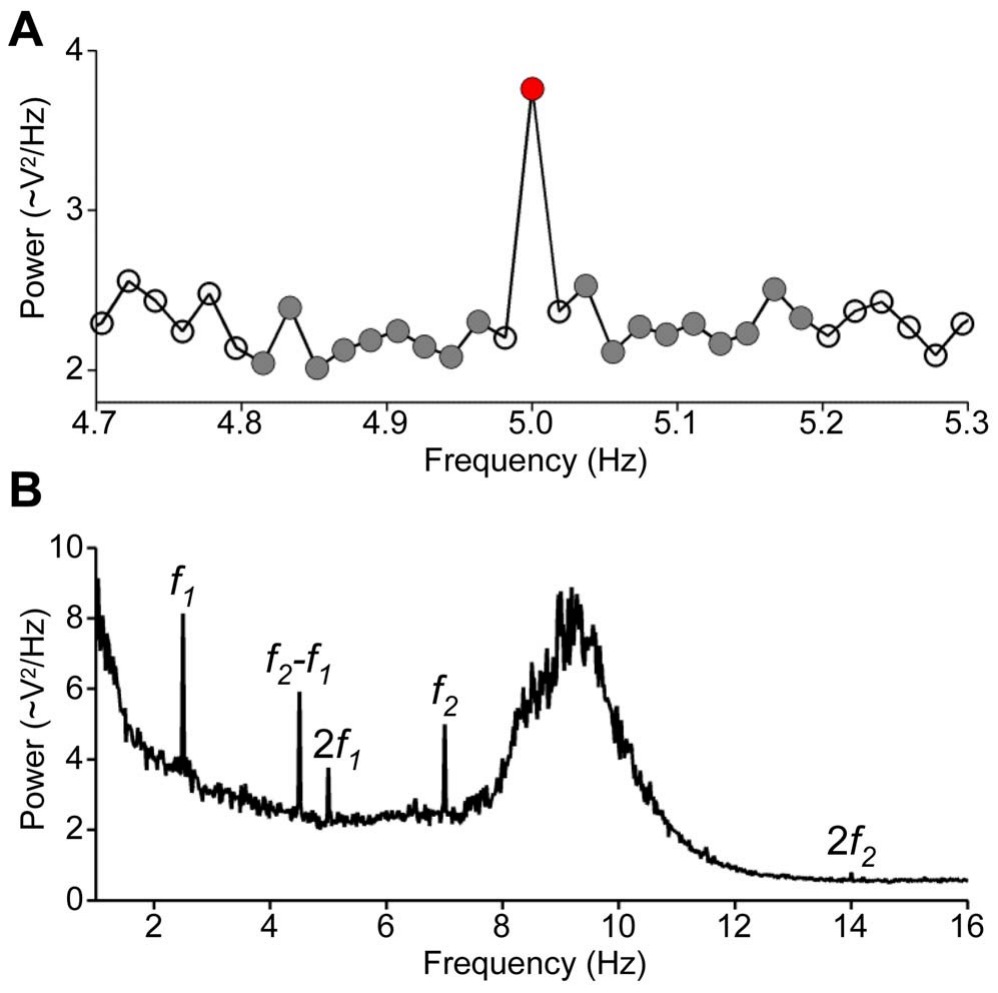
Definition of the neural response. Panel **A** shows group average EEG power spectral density in the dual-AM conditions at a scalp location presumed to reflect auditory-evoked activity (Cz). The magnitude of neural activity to the AMs was assessed using the SSR (which we will refer to as “neural response”), defined as the power ratio of an AM-specific frequency bin (illustrated here for 2f_1_, red circle) to the averaged neighboring bins (baseline, gray circles) minus one. Analyses of shorter time windows (see text, section Time windows of interest) involved fewer baseline bins to avoid overlap between baselines associated with neighboring critical frequencies. Panel **B** provides a larger view of the spectrum shown in panel A. The neural response to the dual AM was significantly stronger than zero for the frequencies of interest (f_1_, f_2_, 2f_1_, 2f_2_, f_2_-f_1_).

Following previous approaches for SSR measurement [57], [58], a normalized measure of the neural response was used; see Figure 3A. This normalized neural response was computed separately for each participant, EEG channel, experimental condition, and frequency of interest as follows. First, for each EEG channel, single-trial EEG power spectral density was estimated by decomposing the channel waveform using the fast Fourier transform. Second, after averaging the single-trial spectra across trials belonging to the same experimental condition, then for each frequency of interest, squared magnitude was extracted for the frequency bin of interest and for “control” bins adjacent to that critical bin (excluding the nearest neighbor on each side of the critical bin to avoid potential leakage effects; see [58]). The adjacent bins were then averaged to define the baseline for the frequency of interest. Finally, for each frequency of interest, the normalized neural response was computed by dividing the power of the frequency of interest by the power of its baseline minus one. This unit-free measure, which we will refer to as “neural response” for simplicity, is unbiased with respect to broad-band signals (e.g. ongoing brain rhythms in the low-frequency range, such as the alpha band visible in Figure 3B), facilitating its comparison across different frequencies and task conditions.

As shown by Figure 3B, initial EEG data exploration for the critical frequencies at scalp location Cz revealed robust neural responses (i.e., significantly larger than zero, the nominal baseline) for the first two harmonics of each AM frequency (statistical group analysis, *t*_13_ = 4.21, 4.29, 3.09, 2.69, *P*<0.0005, 0.0005, 0.005, 0.001 for *f_1_*, *f_2_*, 2*f_1_*, and 2*f_2_*, respectively) and also the beat frequency (*t*_13_ = 3.92, *P*<0.0005), in line with previous observations [34], [35], [37], [38]. No significant response was observed for the control frequencies (all *t*_13_<0.84, *P*>0.22). Based on this initial data quality check (and specifically the significant responses to the first two harmonics) and previous approaches [39], we focused subsequent analyses on the average of the first two harmonics. To that end, we averaged the neural response at *f_1_* with the neural response at 2*f_1_*, separately for each participant, EEG channel, and experimental condition (analogous for the neural responses associated with *f_2_* and 2*f_2_*). In the remainder of this text, we will refer to the averaged harmonics simply as *f_1_* (for the slow modulation) or *f_2_* (for the fast modulation).

#### Extraction of measures of interest

Our first goal was to identify neural activity that followed best the envelope of the individual AMs (temporal AM-frequency representation). To that end, the neural response to the single AM stimuli was computed by averaging the response at *f_1_* in the slow AM condition with the response at *f_2_* in the fast AM condition; this was done separately for each participant and EEG channel. From the resulting frequency-averaged response, EEG channels showing the strongest responses were extracted for each participant. Our main goal was to test whether this temporal AM-frequency representation is sensitive to attention. Therefore, after averaging the selected channels, further attention-specific measures were extracted from the channel-averaged neural responses for each participant: Firstly, the neural response to the targets was extracted by averaging the response at *f_1_* in the slow target condition with the response at *f_2_* in the fast target condition. Secondly, the neural response to the non-targets was extracted analogously by averaging the response at *f_2_* in the slow target condition with the response at *f_1_* in the fast target condition. Thirdly, the neural response to the dual AM under visual attention was extracted by averaging the response at *f_1_* with the response at *f_2_* in the visual target condition. Finally, the effect of AM-selective attention was quantified using a neural dominance index computed by subtracting the response to the non-targets from the response to the targets. Positive values of this index thus indicate stronger neural responses to the target than to the non-target (i.e., neural dominance of the target), whereas negative values indicate the opposite.

#### Time windows of interest

The channel waveforms from which the aforementioned measures were initially extracted spanned either the whole task interval or consecutive portions thereof, both excluding the initial stimulus-onset-response interval (the first 1-s portion of the task interval). The purpose of the latter time-resolved analysis was to inspect slow temporal changes of the relevant measures across the task interval. This analysis was enabled by sliding an 11-s analysis window in 1-s steps across the task interval to create a series of 44 consecutive neural responses associated with partially overlapping time windows. In this way, time series of the aforementioned measures of interested could be generated, and slow changes in these measures could be assessed by fitting a line using least squares and extracting the line slope. This analysis was done separately for each participant, and the resulting individual slopes were then submitted to a statistical group analysis to test whether the slopes differed significantly from zero (i.e., no slow change across the task interval).

The different analysis window durations in the whole-interval analysis and time-resolved analysis inevitably induce different frequency resolutions (0.019 Hz and 0.091 Hz, respectively). To avoid overlap among baselines associated with neighboring frequencies of interest, a different number of baseline bins was used for the two analyses (18 bins and 2 bins, respectively).

#### Correlating neural and behavioral responses

To assess the link between neural and behavioral responses in the auditory task, we first extracted the time series of the neural response; this was done after averaging across the selected channels separately for each trial, each frequency of interest (*f_1_*, *f_2_*), each target condition (slow target, fast target), and each participant. From these single-trial series, a series of neural dominance indices (described above, see section Extraction of measures of interest) was computed; this was done separately for each target condition and each participant. Analogously, we extracted time series of the behavioral response: For each target condition and participant, a series of short-term perceptual dominance indices was computed by sliding an 11-s analysis window in 1-s steps across the behavioral time series (excluding the first 1-s portion of the task interval) and extracting from each window the proportion of time that the participant reportedly perceived the target as dominant. Finally, after concatenating the index series of all trials from the two target conditions, linear dependence between the neural and perceptual indices was assessed for each participant using Pearson's correlation coefficient *r*. Participants' individual correlation coefficients were then submitted to statistical group analysis to test whether *r* differed significantly from zero (*i.e*., no correlation) after excluding data from four participants (P11-P14) who showed insufficient variance in their behavioral response (Figure S2).

### Statistical analysis

The relevant measures that were obtained from each participant (the aforementioned measures of interest, neural and perceptual indices, Fisher transform of *r*, and line slope) were submitted to group analyses using nonparametric statistical tests [59]. Condition labels were randomly shuffled for each participant and a paired *t*-test statistic was computed from the shuffled data. This procedure was iterated 5000 times to create a distribution of permutation-based *t*-statistics. A permutation-based *P*-value was computed as the proportion of iterations for which the permutation-based *t*-statistic was larger than the *t*-statistic obtained from the original data (reported in section Results). The significance criterion α was set to 0.05.

## Results

### Behavioral results

**Figure 4:**
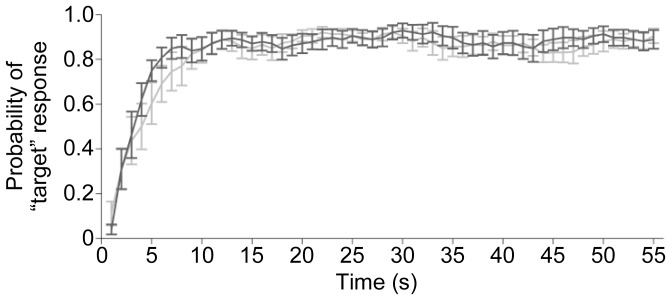
Behavioral results. The plot shows the average probability of perceiving the target as dominant, as a function of time separately for the slow target (dark gray) and fast target (light gray). Error bars represent s.e.m. across all participants. See Figure S2 for single-subject data.

Figure 4 shows the proportion of trials for which listeners reported perceiving the target as dominant, plotted as a function of time (see Figure S2 for single-subject data). The target dominated listeners' percept for 82±3% of the auditory task time (perceptual dominance index, mean ± s.e.m. across participants) with no significant difference between slow and fast targets (*t*_13_ = 0.74, *P* = 0.47). The initial target percept appeared to evolve gradually after stimulus onset, which has also been observed in studies on auditory streaming [60]. An analysis of curve slopes (see section Behavioral data analysis) revealed that this perceptual build-up finished approximately within the first 13 seconds. Excellent performance was observed in the visual task (*d*' = 4.52±0.2, mean ± s.e.m. across participants), suggesting that participants paid attention to the visual stimuli.

### Temporal AM-frequency representation in cortex

**Figure 5:**
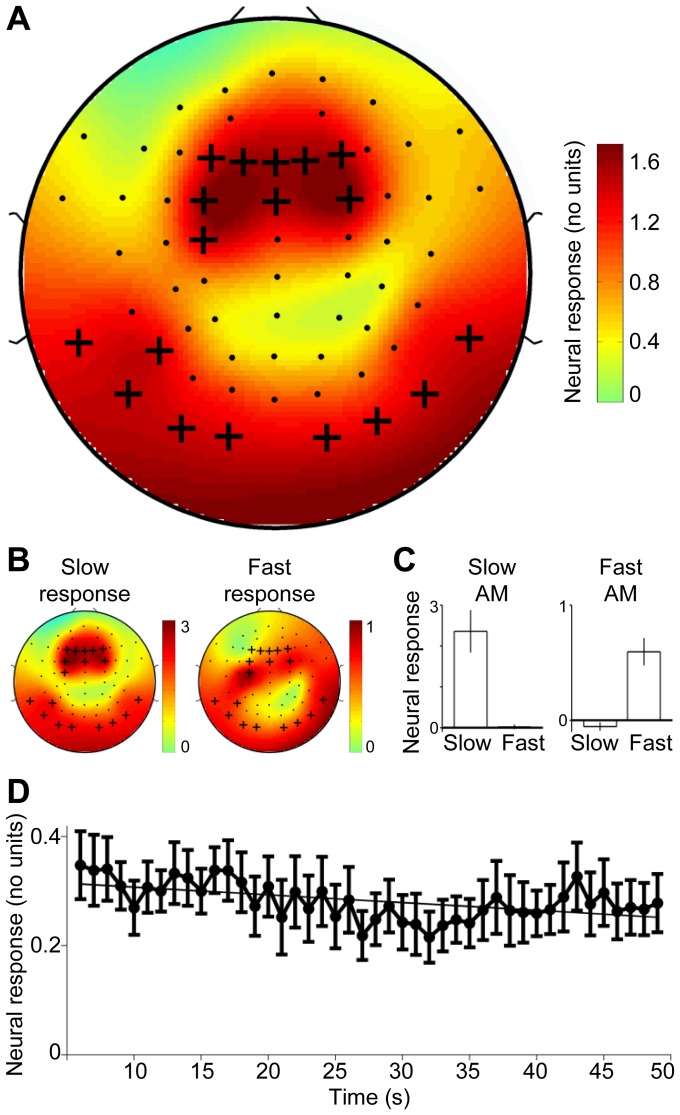
Temporal AM-frequency representation in cortex. Panel **A** shows the scalp distribution of the group average neural response to the single AMs, suggesting a neural origin in AC. EEG channels for which neural activity followed best the individual AM frequencies were selected (crosses), averaged, and further tested for attention-related effects (see Figure 6). See Figure S3 for single-subject data. Panel **B** shows scalp topographies analogously to panel A, but separately for the slow response and the fast response. Panel **C** shows the magnitude of the neural response in the slow AM condition (left plot) and fast AM condition (right plot), averaged across the channels selected in panel A. Within each plot, the left bar corresponds to the slow response (i.e., at *f_1_*) and the right bar corresponds to the fast response (i.e., at *f_2_*). Error bars represent s.e.m. across all participants. Panel **D** shows the magnitude of the neural response over time averaged across the channels selected in panel A. Error bars represent s.e.m. across all participants. The units are the same as in panel A, and the lower magnitudes result from using a shorter analysis window (see section Time windows of interest). The fitted line exhibits a significant negative slope, indicating that this response adapted across the task interval.

To extract neural activity that follows best the individual AM frequencies, we first explored the scalp distribution of the average neural response to the single AM stimuli. Consistent with previous data [58], Figures 5A–C show the strongest responses in fronto-central and temporo-posterior scalp regions (see red regions/crosses in Figure 5A; channels Fz, F1, F2, F3, F4, FCz, FC3, FC4, C3, TP9, TP10, P7, P9, P10, PO9, PO10, O9, O10; see Figure S3 for single-subject data). These regions, which we considered to reflect stimulus phase-locked activity of neuronal populations in bilateral AC based on previous EEG source analyses [40], [61], [62], were then selected for channel-averaged analyses testing for attention-related effects. Averaging across 10, 20, or 50 channels yielded similar results, suggesting that the specific number of selected channels played little role as in related studies [12], [13]. The time-resolved analysis of the extracted neural response (i.e., the time course of the average neural response to the single AM stimuli, averaged across the selected channels) revealed that our single AM stimuli evoked robust phase-locking throughout the task interval (neural response>0, Figure 5D). Fitting a line and analyzing the line slope revealed adaptation, i.e., the neural response became weaker across the task interval (line slope <0: *t*_13_ = −1.79, *P* = 0.049, Figure 5D), consistent with previous ideas [37].

### Effect of AM-frequency selective attention

**Figure 6:**
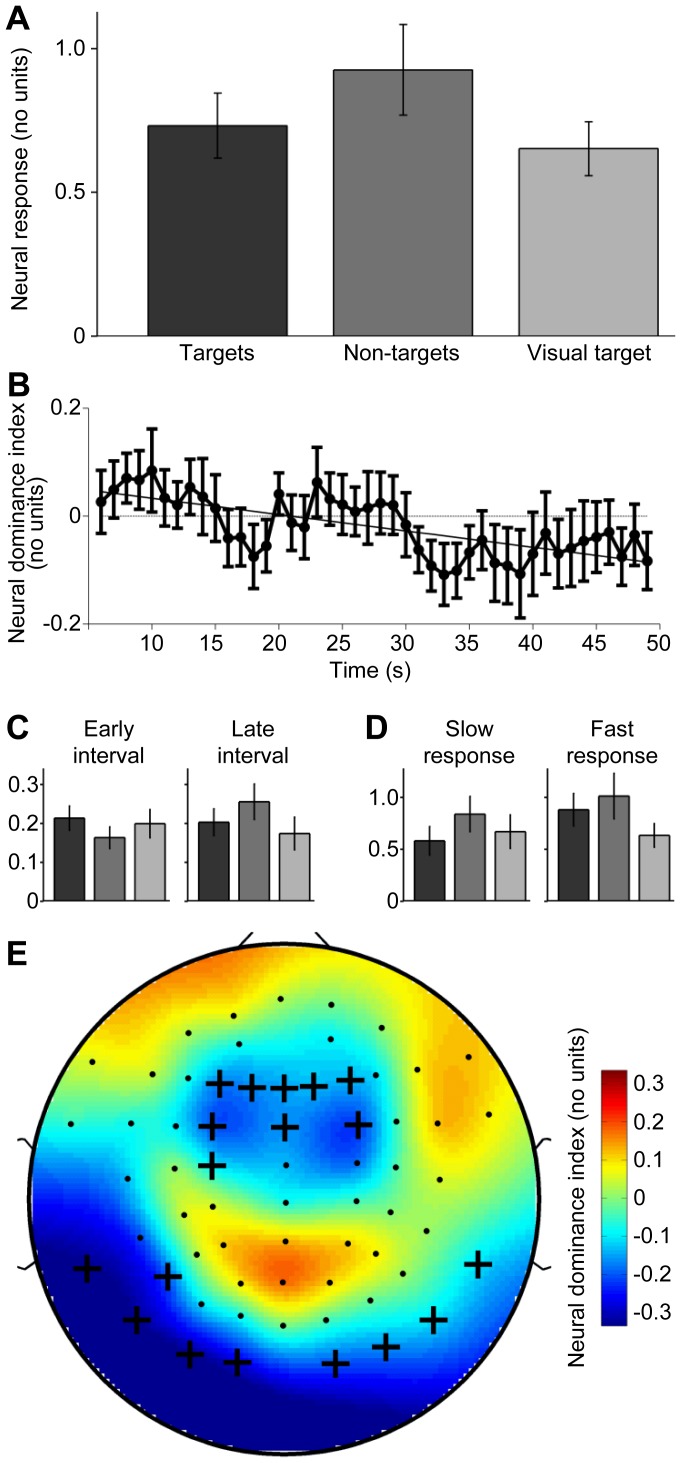
Effects of attention on AM representation in cortex. Panel **A** shows the channel-averaged neural response to the dual AM stimulus in the different attention conditions. Overall, the response was significantly stronger for the non-targets than the targets. Furthermore, the response was substantially stronger during auditory attention than visual attention. Error bars represent s.e.m. across all participants. See Figure S4 for single-subject data. Panel **B** shows the time course of the neural dominance index (defined as the neural response to targets minus the neural response to non-targets) and fitted linear trend (oblique line), indicating that the observed dominance of non-targets (panel A) arose mostly late during the task interval. Error bars represent s.e.m. across all participants. Panel **C** shows time-averaged neural responses as in panel A limited to the initial 15-s interval (left) and the final 15-s interval (right), illustrating the change in neural dominance from early to late interval. The units are the same as in panel A, and the lower magnitudes result from using a shorter analysis window (see section Time windows of interest). Panel **D** also shows neural responses as in panel A, but separately for the slow neural response (left) and the fast neural response (right), revealing overall similar patterns. Panel **E** shows the spatial distribution of the neural dominance index. Blue and red hue indicates neural dominance of the non-targets and targets, respectively. Crosses indicate the channels from which the results in the other panels were obtained (same as in Figure 5A).

Figure 6 illustrates our main result, the effect of AM-selective attention on the channel-averaged neural response. Whole-interval analysis revealed that this response differed significantly for targets vs. non-targets (*t*_13_ = −2.38, *P* = 0.025). Surprisingly, the response to *non-targets* was stronger (Figure 6A, neural dominance index <0; see Figure S4 for single-subject data). No significant effect was observed for the stimulus-unrelated control frequencies (*t*_13_ = 1.73, *P* = 0.11). These results thus contradict our hypothesis of sustained AM-selective attentional enhancement.

In the following four analyses, we explored this result in more detail, i.e., for shorter time intervals (Figures 6B, C), in relation to listeners' perceptual reports (Figure S2), for separate AM frequencies (Figure 6D), and at individual scalp locations (Figure 6E).

Firstly, plotting the neural dominance index over time revealed negative values mostly during the late portions of the task interval (Figure 6B), which suggests that the observed effect arose only after some delay. This notion was supported by fitting a line to the time series and analyzing the line slope, which showed that the “negative” effect built up slowly (fitted line slope <0: *t*_13_ = −1.78, *P* = 0.037). Notably, the initial 15-s interval showed exclusively positive values. Within this early interval, the neural response exhibited a pattern across attention conditions (Figure 6C, left) that agrees qualitatively with our initial hypothesis of AM-selective attentional enhancement; however, the difference between responses to targets vs. non-targets was not statistically significant (*t*_13_ = 1.15, *P* = 0.14). For reference, Figure 6C (right) shows the average neural response during the final 15-s interval (targets vs. non-targets: *t*_13_ = −1.16, *P* = 0.12).

Secondly, correlation analysis of neural and behavioral dominance indices revealed no significant result (*t*_9_ = −1.54, *P* = 0.082). However, the same analysis applied to a dataset that excluded the initial perceptual build-up interval (this interval contained no or only few changes in perception; see Behavioural results) revealed weak but significant coupling (average *r* = −0.037 <0: *t*_9_ = −1.78, *P* = 0.048): the longer the target dominated the listener's percept during the analysis interval, the more the non-target dominated the average neural response during this interval. No statistically significant correlation was observed for the stimulus-unrelated control frequencies (*t*_9_ = −0.99, *P* = 0.17).

Thirdly, the neural response exhibited a similar pattern for each AM frequency (Figure 6D), i.e., there was no statistically significant interaction (*f_1_*/*f_2_* × target/non-target: *t*_13_ = 0.77, *P* = 0.24), suggesting that slow targets and fast targets contributed similarly to the overall effect.

Finally, plotting the neural dominance index separately for each channel revealed a spatial distribution across the scalp highly complementary to that of the average response to the single AM stimuli (*r* = −0.80; compare Figure 6E vs. Figure 5A), thus providing no indication that neural generators outside AC were the source of the effect.

### Effect of auditory vs. visual attention

Figure 6 further illustrates the effect of auditory attention relative to visual attention. Whole-interval analysis revealed that the neural response to the dual AM was significantly stronger in the auditory task than the visual task (*t*_13_ = 2.22, *P* = 0.019; Figure 6A; see Figure S4 for single-subject data), consistent with findings based on more rapidly modulated sounds [15], [16], [17]. As shown by Figure 6A, this enhancement relative to the visual task was driven mostly by the response to non-targets (*t*_13_ = 2.71, *P* = 0.0024) and to a smaller, non-significant extent by the response to targets (*t*_13_ = 1.05, *P* = 0.15). No effect was observed for the stimulus-unrelated control frequencies (*t*_13_ = −1.35, *P* = 0.90).

### Beat frequency representation in cortex

**Figure 7:**
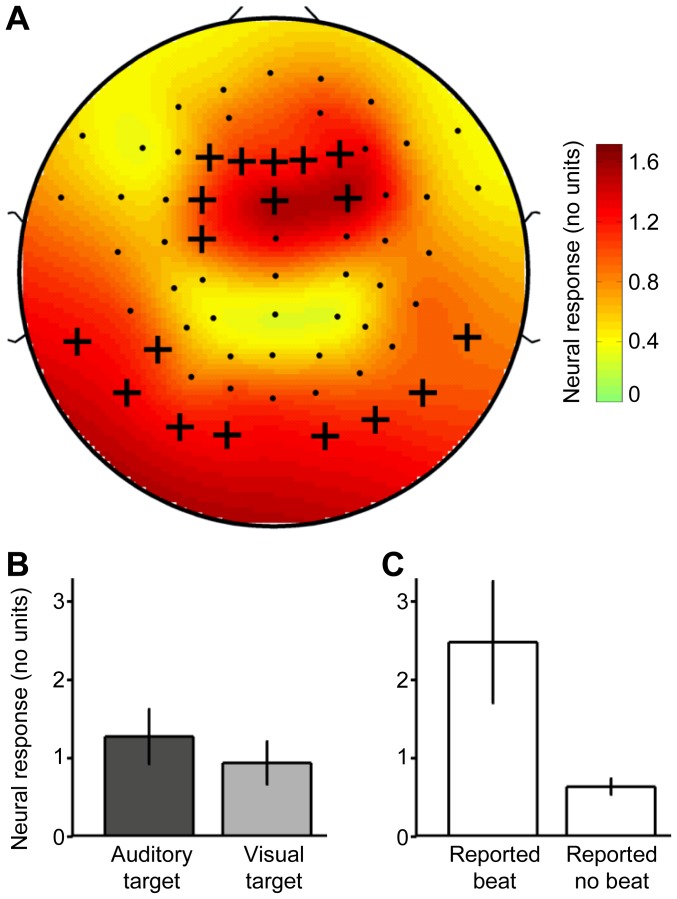
Beat frequency representation in cortex. Panel **A** shows the scalp distribution of the group average neural response associated with the beat frequency in the dual-AM conditions. Crosses indicate the channels from which the results in the other panels were obtained (same as in Figure 5A). Panel **B** shows the channel-averaged neural response associated with the beat frequency in the different attention conditions. The response was significantly stronger during auditory attention than visual attention. Error bars represent s.e.m. across all participants. Panel **C** shows the channel-averaged neural response associated with the beat frequency in the dual-AM conditions, separately for participants who reported hearing a beat and participants who reported hearing no beat. The response was significantly stronger for participants reporting a beat. Error bars represent s.e.m. across four and ten participants (left and right column respectively).

Exploratory analysis of the neural response associated with the beat frequency (obtained after averaging the dual-AM conditions) revealed results similar to those obtained for the two AM-stimulus frequencies: The scalp distribution was highly similar (*r* = 0.86) to that observed before (compare Figures 7A, 5A). Channel-averaged analysis further revealed a positive effect of the auditory tasks compared with the visual task as before (*t*_13_ = 1.84, *P* = 0.048; Figure 7B). Finally, group comparison revealed that participants who reported hearing a third beating rhythm in the auditory task (participants P1, P7, P10, P11) produced stronger neural responses at the beat frequency (normalized with respect to the average of slow response and fast response) than participants who reported not hearing such a rhythm (Wilcoxon-Mann-Whitney Test, *U* = 38, *P* = 0.0040); see Figure 7C.

## Discussion

Previous studies have shown that selective attention to an AM sound enhances the SSR evoked by the AM of that sound, compared with the SSR evoked by a competing, unattended AM sound with distinct tone frequency or location [10], [11], [12], [13]. The main goal of our study was to test if similar selective attentional enhancement occurs in the absence of location and pitch cues, i.e., when attention is focused exclusively and continuously on a specific ongoing AM.

We observed an enhancement of temporal AM-frequency representations likely located in AC (as measured by the SSR) during sustained auditory attention relative to visual attention, consistent with previous findings [15], [16], [17], [18]. In contrast to other findings based on shorter intervals [10], [11], [12], [13], sustained AM-selective attention produced overall stronger neural enhancement for the *non-target* AM than the target AM. Thus, overall, this main result does not support the notion of sustained AM-selective attentional enhancement in AC.

We found further that the neural dominance of the non-targets evolved rather slowly: the longer listeners had attempted to hear out a specific AM frequency, the less that AM frequency dominated the temporal AM representation. Because our AMs were invariant both within and between attention conditions, this adaptive effect of sustained AM-selective attention must be attributed to non-acoustic factors, such as the short-term history of the listener's perceptual state or potential transient learning effects [63] that influenced top-down attentional engagement. Perceptual AM-specific adaptation is known to apply to a range of AM frequencies including the ones used here [44], [45], [46], [47]. A candidate mechanism underlying this perceptual phenomenon is AM-specific neural adaptation in AC, which has been observed in monkeys using AMs similar to the ones used here [64]. In our study, the neural response to single AMs attenuated significantly over the task interval (Figure 5D), showing that continuous exposure to these AMs rendered them less dominant in cortex. Furthermore, several participants in our study reported informally that they perceived the target as slowly fainting until the percept switched toward the competing non-target, after which the desired target became more perceivable again. Considering findings of carrier frequency- and pitch-specific adaptation in AC [65], [66], these observations suggest that our results reflect AM-specific adaptation that comprised response enhancement during sustained AM-selective attention. A possible purpose of this putative AM-specific adaptation could be to bias temporal processing in AC toward AM frequencies outside the listener's focus of sustained attention in order to support the auditory system in keeping track of task-irrelevant sound features [67] such as different AM frequencies.

Closer inspection of the initial sound interval revealed a trend that fits well with the previous findings of attentional response enhancement during this interval [10], [11], [12], [13]. The fact that we could not detect a more robust (statistically significant) AM-selective attentional response enhancement during this interval may be due to the rather limited number of data points (i.e., few trials related to the long duration of our stimuli). Another potential explanation is that, in the absence of location and pitch cues, AM-selective attentional enhancement applies predominantly to non-temporal AM-frequency representations that were not captured by our SSR measure, for example, spatially segregated AM frequency channels (see Introduction) whose outputs exhibit no phase-locking. A final potential explanation is that robust selective attentional enhancement of temporal AM representations in AC requires the competing sounds to be distinguishable based on multiple distinct features, rather than AM frequency alone. In other words, the lack of a robust effect during the initial portion of our stimulus may be due to the absence of pitch and location cues, which compromised listeners' ability to perceptually segregate the competing AMs and thereby compromised attentional response enhancement. Further research is needed to disentangle these possibilities.

Conclusively, selective attentional cortical gain control based on AM frequency seems to operate adaptively under sustained attention and within an early interval. Furthermore, this mechanism seems to benefit from the presence of multiple distinct sound features, e.g., when the competing sounds differ not only in their AM, but also their location and pitch. These additional distinct features may facilitate performance in sustained attention tasks by allowing listeners to shift their attentional focus across these features, thereby enabling the feature-based cortical gain control mechanism to overcome adaptation to a specific feature.

A noteworthy side finding is that the cortical interaction of the competing AM frequencies (i.e., the beating frequency), which was already observed in a previous study [33], was enhanced both during auditory attention (compared with visual attention) and in listeners who reported hearing well the beating (compared with listeners who reported hearing no beating). These observations support the earlier suggestion [35] that the strength of the auditory cortical representation of the beating pattern determines the perceived salience of that beat.

## Supporting Information

Figure S1 **ICA-based artifact reduction.** The figure shows for each participant (P1-P14) the centroid power spectral density, the centroid weights (scalp topography), and the IC weights underlying the centroid (from left to right). Furthermore, for each participant, the upper half shows data (centroid spectrum, centroid weights, individual IC weights) that were considered brain activity, and the lower half shows data considered artifacts. On average, ICs labeled as brain activity showed a more marked and dipole-like scalp topography (see centroid weights) and clearer harmonics in the frequency range of our AMs (see centroid spectrum), compared with ICs labeled as artifacts.
(JPG)

Figure S2 **Behavioral results per participant.** Analogous to Figure 4, the time series show for each participant the probability of perceiving the target as dominant, separately for the slow target (dark gray) and fast target (light gray).
(JPG)

Figure S3 **Temporal AM-frequency representation in cortex per participant.** Analogous to Figure 5A, the plots show for each participant the scalp distribution of the average neural response to the single AMs. Crosses indicate the channels from which the data in Figure S3 were obtained (same as in Figure 5A).
(JPG)

Figure S4 **Effects of attention on AM representation in cortex per participant.** Analogous to Figure 6A, the plots show for each participant the channel-averaged neural response to the dual AM stimulus in the attention conditions.
(JPG)
