# Supplementary figures and images for: Sustained Selective Attention to Competing Amplitude-Modulations in Human Auditory Cortex
Source: PLoS One. 2014 Sep 26;9(9):e108045. doi: 10.1371/journal.pone.0108045 (PMC4178064; doi:10.1371/journal.pone.0108045)

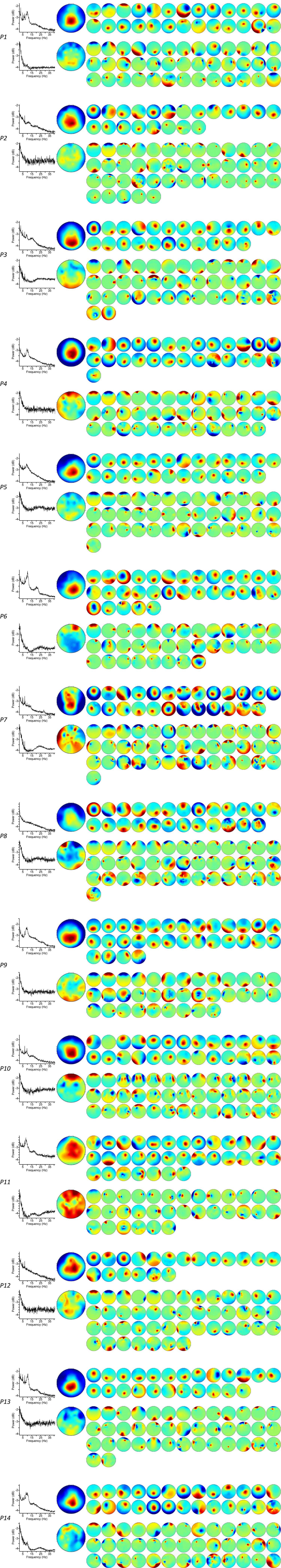

Supplement: Figure S1 — ICA-based artifact reduction. The figure shows for each participant (P1-P14) the centroid power spectral density, the centroid weights (scalp topography), and the IC weights underlying the centroid (from left to right). Furthermore, for each participant, the upper half shows data (centroid spectrum, centroid weights, individual IC weights) that were considered brain activity, and the lower half shows data considered artifacts. On average, ICs labeled as brain activity showed a more marked and dipole-like scalp topography (see centroid weights) and clearer harmonics in the frequency range of our AMs (see centroid spectrum), compared with ICs labeled as artifacts. (JPG) [file pone.0108045.s001.jpg]

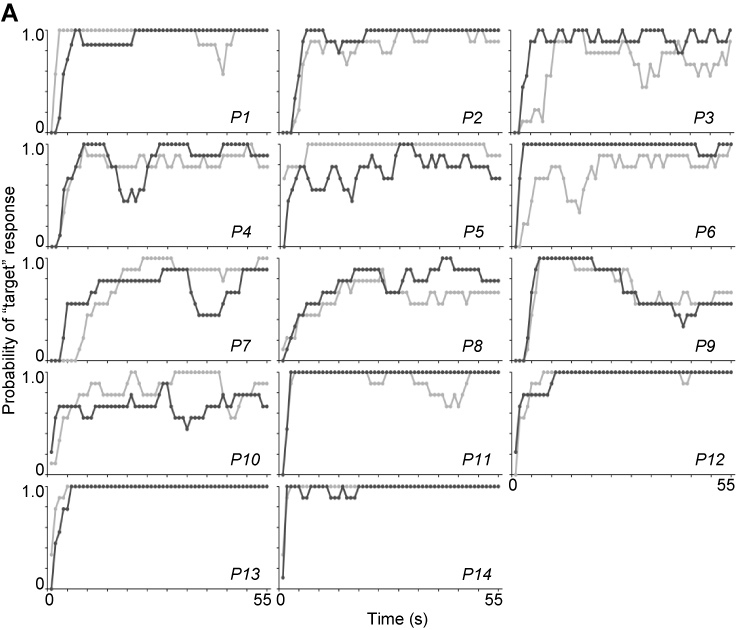

Supplement: Figure S2 — Behavioral results per participant. Analogous to Figure 4, the time series show for each participant the probability of perceiving the target as dominant, separately for the slow target (dark gray) and fast target (light gray). (JPG) [file pone.0108045.s002.jpg]

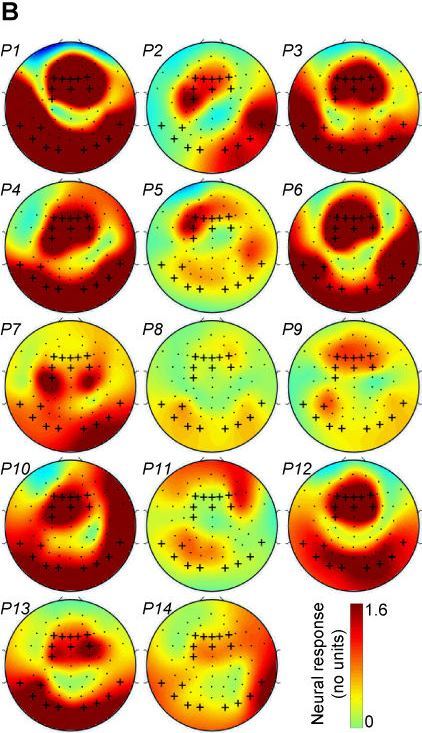

Supplement: Figure S3 — Temporal AM-frequency representation in cortex per participant. Analogous to Figure 5A, the plots show for each participant the scalp distribution of the average neural response to the single AMs. Crosses indicate the channels from which the data in Figure S3 were obtained (same as in Figure 5A). (JPG) [file pone.0108045.s003.jpg]

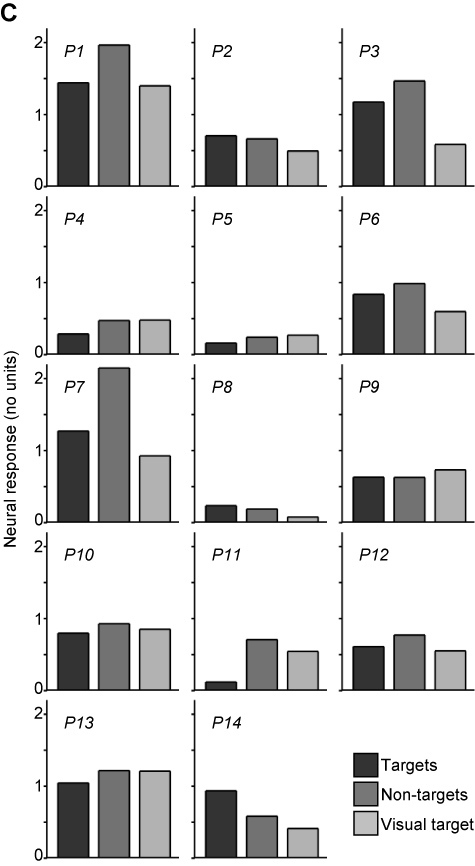

Supplement: Figure S4 — Effects of attention on AM representation in cortex per participant. Analogous to Figure 6A, the plots show for each participant the channel-averaged neural response to the dual AM stimulus in the attention conditions. (JPG) [file pone.0108045.s004.jpg]
